# Deep learning–based high-throughput phenotyping for tiller quantification in interspecific bentgrass hybrids using YOLOv8

**DOI:** 10.3389/fpls.2026.1810220

**Published:** 2026-05-01

**Authors:** Dennis W. Ferm, Yonghyun Kim, Jinyoung Y. Barnaby

**Affiliations:** Floral and Nursery Plants Research Unit, U.S. National Arboretum, Agricultural Research Services, United States Department of Agriculture, Beltsville, MD, United States

**Keywords:** bentgrass hybrids, computer vision, deep learning, faster R-CNN, high-throughput phenotyping, tiller quantification, turfgrass breeding, YOLOv8

## Abstract

**Introduction:**

Tiller production is a critical determinant of turfgrass canopy density and plant performance, yet manual tiller counting is too labor-intensive for large breeding programs.

**Methods:**

To address this limitation, we evaluated 770 plants from an interspecific bentgrass hybrid population and developed three automated approaches for tiller quantification: a classical edge-based segmentation pipeline and two deep-learning models, Faster R-CNN and YOLOv8. Using a large annotated image dataset, we assessed each method’s accuracy, robustness under occlusion, and computational efficiency.

**Results:**

Although two-stage detectors are often expected to provide superior precision for complex plant structures, the one-stage YOLOv8 model achieved the highest accuracy (R² = 0.97) and processed images substantially faster than Faster R-CNN, while both the edge-based method and Faster R-CNN showed reduced performance in dense canopies.

**Discussion:**

These findings demonstrate that recall-oriented one-stage detection can outperform more complex two-stage models for phenotyping tasks involving fine, highly occluded structures. The resulting workflow provides a reliable, high-throughput solution for generating biologically meaningful tiller counts and offers a transferable framework for integrating image-derived phenotypes into genetic analyses and breeding pipelines across grass species.

## 1 Introduction

Improving crop cultivars with enhanced productivity and resilience to environmental stress remains a central objective of modern plant breeding programs. In grass species used for turf and forage systems, canopy density, biomass accumulation, and seed yield are strongly influenced by tiller production, making tiller number a critical quantitative trait for selection and genetic improvemen. Although molecular breeding, genomic selection, and high-throughput genotyping have advanced rapidly, genetic gain is still constrained by phenotyping, as complex morphological traits take considerable time and labor to measure (Cabrera-Bosquet et al., 2012; Das et al., 2019; Song et al., 2021). Tiller quantification exemplifies this bottleneck: manual counting is destructive, time-consuming, and prone to human fatigue and subjective variability, especially when hundreds or thousands of plants must be evaluated within narrow seasonal windows. Developing scalable, reproducible phenotyping approaches that preserve measurement accuracy while dramatically increasing throughput has therefore become a priority in turfgrass and cereal breeding.

Creeping bentgrass (Agrostis stolonifera L.) and colonial bentgrass (Agrostis capillaris L.) are widely cultivated turfgrass species valued for their contrasting yet complementary agronomic characteristics. Creeping bentgrass exhibits dense stoloniferous growth, fine leaf texture, and tolerance to extremely low mowing heights, making it ideal for high-performance turf surfaces but often requiring intensive management (Beard and Green, 1994; Amundsen and Warnke, 2014). Colonial bentgrass typically provides greater drought tolerance, improved disease resistance, and more upright tiller architecture, traits advantageous for lower-input systems (Beard and Green, 1994; DaCosta and Huang, 2006). Interspecific hybridization between these species offers a promising route to combine productivity with environmental resilience, but effective selection within hybrid populations depends on reliable, high-throughput phenotyping of traits such as tiller number, canopy structure, and biomass accumulation.

Traditional tiller counting relies on destructive harvesting or meticulous manual separation of plant material, both of which are impractical for large breeding populations. Individual bentgrass plants can produce hundreds to more than one thousand tillers, and field densities may exceed tens of thousands per square meter (Beard and Green, 1994; Amundsen and Warnke, 2014). These biological realities amplify measurement difficulty and introduce variability associated with human error. As breeding programs increasingly integrate genomic tools and quantitative trait mapping, the inability to rapidly and accurately measure tiller production has become a limiting factor in translating genetic potential into realized agronomic improvement (Furbank and Tester, 2011; Araus and Cairns, 2014; Yang et al., 2020). This disparity between genotyping efficiency and phenotyping capacity underscores the need for automated, image-based solutions that deliver both scale and reproducibility (Jiang and Li, 2020; Ubbens et al., 2025).

Advances in computer vision and deep learning have transformed image-based plant phenotyping by enabling non-destructive, high-throughput assessment of structural traits. Convolutional neural networks have shown strong performance in detecting reproductive organs such as tassels, spikes, pods, and plant stands (Lu et al., 2015; Rawat and Wang, 2017; Sadeghi-Tehran et al., 2019; Alzadjali et al., 2021; Khaki et al., 2022). However, these successes often involve relatively large and visually distinct targets. Bentgrass tillers present a markedly different challenge: they are thin, filamentous, and densely clustered near the crown, producing heavy occlusion that confounds both classical segmentation algorithms and modern object-detection architectures. In canopy-level imagery, individual tillers are rarely separable due to limited contrast, structural overlap, and shadowing (Ghassemi et al., 2025). As a result, approaches successful in cereal crops do not directly translate to turfgrass species characterized by fine-scale morphological complexity and extreme instance density.

Object-detection frameworks commonly used in agricultural computer vision fall into two major architectural paradigms. Two-stage detectors, such as Faster R-CNN, generate region proposals prior to classification and typically achieve high localization precision but at substantial computational cost (Ren et al., 2015; He et al., 2017; Carranza-García et al., 2020). Single-stage detectors, such as the YOLO family, perform localization and classification simultaneously, prioritizing detection speed and recall and enabling real-time inference in complex visual environments (Redmon et al., 2016; Bochkovskiy et al., 2004; Ultralytics, 2023). Although two-stage models often report higher mean average precision (Hui, 2018; Cai and Vasconcelos, 2018), counting accuracy in crowded scenes depends more on minimizing missed detections than on perfectly delineating boundaries (Mohammed, 2025). This distinction is particularly relevant for small, overlapping plant structures like bentgrass tillers, where instance separation—not boundary precision—is the dominant challenge. Consequently, it is not obvious *a priori* whether a two-stage or one-stage architecture is better suited for dense, occluded tiller detection, making a direct comparison scientifically meaningful.

Despite rapid growth in deep-learning applications for plant phenotyping, automated tiller quantification in bentgrass has not been systematically evaluated under controlled imaging conditions designed to mitigate occlusion and maximize separability. Prior work on tiller estimation has focused primarily on cereal crops and often relies on side-view imaging, LiDAR, UAV-based canopy imaging, or density-map regression (Li et al., 2018; Singh et al., 2022; Kinose et al., 2022). Although UAV platforms have shown promise for estimating tiller density and biomass in row crops, their resolution is sufficient for bentgrass: fine tillers, narrow leaf blades, and low contrast against soil make individual tillers in turfgrass canopies. These limitations highlight the need for a reproducible, high-throughput workflow tailored to species with extremely fine morphology, where aerial phenotyping cannot achieve the precision required for genetic mapping. A pipeline capable of delivering accurate instance-level tiller counts-under imaging conditions in which individual organs can be clearly separated-would alleviate a persistent phenotyping bottleneck and enable population-scale trait analysis, quantitative trait locus (QTL) mapping, and marker-assisted selection aimed at improving vigor, yield potential, and stress resilience.

To address these challenges, the present study employs a ground-based, laboratory-controlled imaging approach in which tillers were manually clipped and photographed against standardized backgrounds to ensure maximal scalability. The objectives were to (i) develop an automated tiller quantification workflow using controlled imaging and preprocessing conditions, (ii) compare a classical edge-based segmentation pipeline with two deep-learning object-detection architectures—Faster R-CNN (two-stage) and YOLOv8 (one-stage)—to evaluate how architectural differences influence counting accuracy under occlusion, and (iii) characterize population-level tiller variation within an interspecific bentgrass hybrid population to support downstream genetic analysis. By integrating computer vision, deep learning, and quantitative breeding objectives, this work addresses a longstanding phenotyping limitation in turfgrass research and establishes a methodological framework suited forspecies in which UAV-based phenotyping does not provide adequate resolution for organ-level quantification.

## 2 Materials and methods

### 2.1 Plant materials and experimental design

A total of 770 individual plants were evaluated, consisting of 254 F_1_ hybrid progeny derived from an interspecific cross between creeping bentgrass (*Agrostis stolonifera* ‘Providence’) and colonial bentgrass (*Agrostis capillaris* ‘BCD’), three clonal replications for F_1_ lines where available, and the two parental controls. The parental genotypes were selected for their contrasting agronomic characteristics: ‘Providence’ typically exhibits high tiller production and yield potential but greater susceptibility to drought and disease, whereas ‘BCD’ generally produces fewer tillers but demonstrates improved drought tolerance and disease resistance. This cross was developed to combine the productivity of creeping bentgrass with the environmental resilience of colonial bentgrass (Kim et al., 2024). Plants were established in 5-gallon pots embedded within field soil at the Beltsville Agricultural Research Center (BARC) North Farm, Beltsville, Maryland, USA. Pot confinement limited lateral stolon spread and enabled more accurate estimation of genotype-level yield potential while maintaining field-relevant environmental conditions. Pots were arranged in a randomized layout in fall 2022 and maintained under standard irrigation and cultural practices throughout the growing season. All plants were destructively harvested between August and September 2023 at the end of the growth cycle to obtain tiller clippings for imaging and manual counting. Each sample was barcoded to ensure traceable identification throughout image acquisition and data processing.

**Figure 1:**
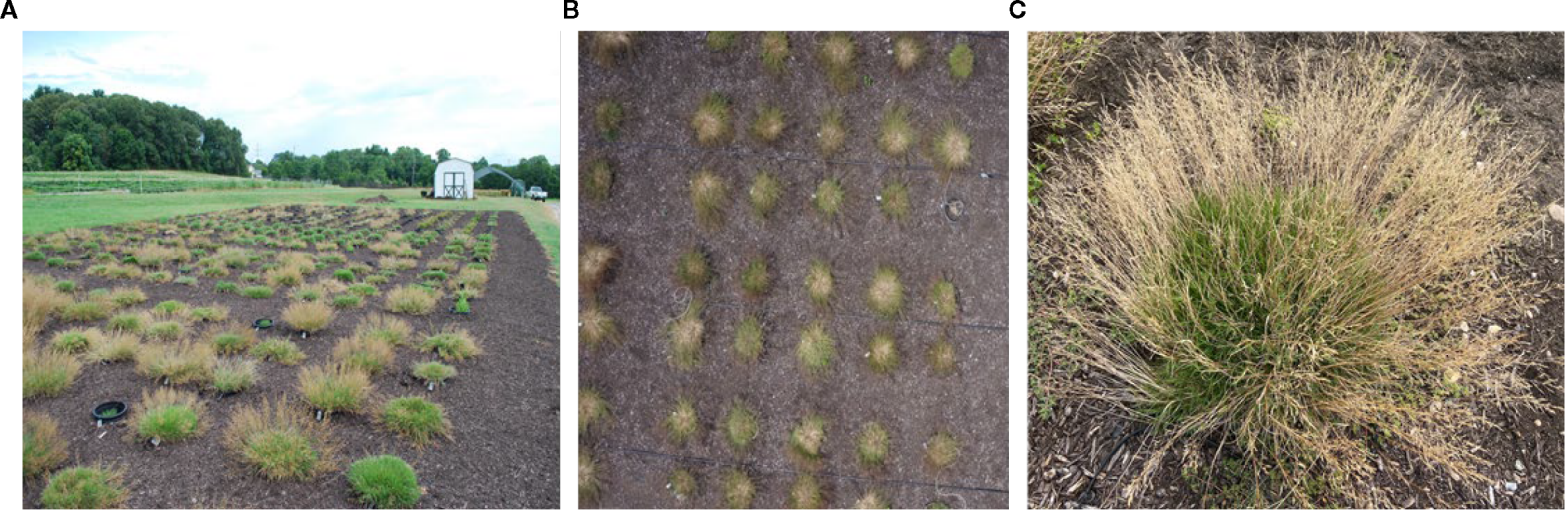
Field-to-sensor perspectives of the interspecific bentgrass hybrid population. **(A)** Field image showing dense, uniform turf canopy produced by hybrid lines; **(B)** UAV RGB image highlighting the difficulty of resolving thin, filamentous tillers against soil/canopy background; **(C)** ground-level image of a single hybrid line showing fine tiller morphology and visual occlusion that complicates automated in-situ counting.

Tiller number is a critical yield indicator in grass species, and many lines in the interspecific bentgrass hybrid population produced more than 1,000 tillers per plant (**Figure 1A**), making manual counting impractical for high-throughput breeding. Although deep learning approaches have successfully quantified reproductive structures such as corn tassels and wheat spikes from RGB imagery, bentgrass tillers present a distinct challenge due to their thin, filamentous morphology and dense overlap near the crown. These characteristics make individual tillers difficult to separate from soil or canopy background in UAV imagery (**Figure 1B**) and even in higher-resolution ground-level images (**Figure 1C**). To overcome these constraints, we developed an automated counting workflow based on harvested clipped tillers and evaluated three approaches: a classical edge-based segmentation pipeline implemented in OpenCV, and two convolutional neural network (CNN) object detectors, Faster R-CNN and YOLOv8.

### 2.2 Tiller clipping preparation and image acquisition

**Figure 2:**
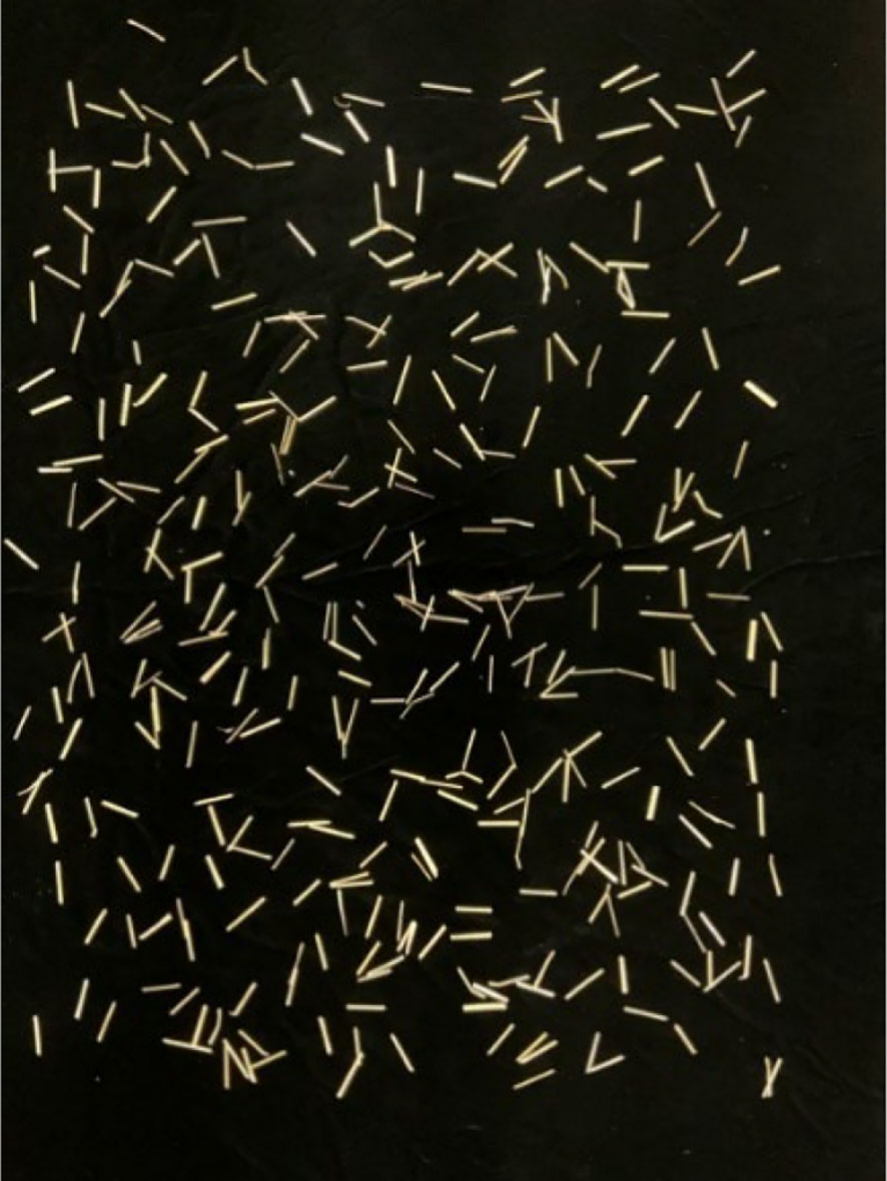
Representative raw image of a tiller sample illustrating common occlusion. Individual tillers frequently overlap or cross, producing partial edge loss and merged silhouettes that confound instance separation in both classical and learned detectors. This visual context motivates standardized clipping, spreading, and background selection for imaging.

To standardize visual input and minimize classification noise during automated detection, preliminary trials compared clipping lengths of 1.0, 1.5, and 2.0 cm. Clippings ≤1.5 cm produced elevated false detections because small debris such as seed fragments and leaf tips were frequently misclassified as tillers. Therefore, a uniform clipping length of 2.0 cm was adopted for all samples. Harvested tillers were evenly distributed on a black velvet background selected for its light-absorbing properties and strong contrast with green plant tissue, improving foreground–background separation and reducing segmentation artifacts. Images were captured using an Apple iPad Pro (6th generation) equipped with a 12-megapixel wide-angle camera (29-mm focal length, f/1.8 aperture) mounted on a fixed overhead platform positioned 45 cm above the sample surface. All images were acquired in a controlled indoor laboratory environment under uniform ambient lighting to minimize illumination variability and shadow formation. Images were stored in JPEG format at a resolution of 3024 × 4032 pixels (~300 dpi), covering an approximate field of view of 30 × 40 cm (**Figure 2**). Manual tiller counts were conducted immediately following image capture by visual separation and served as the ground-truth reference for all model evaluations. Although labor-intensive, manual counting provides the most reliable benchmark for validating automated instance-level detection approaches.

### 2.3 Classical edge-based segmentation pipeline

**Figure 3:**
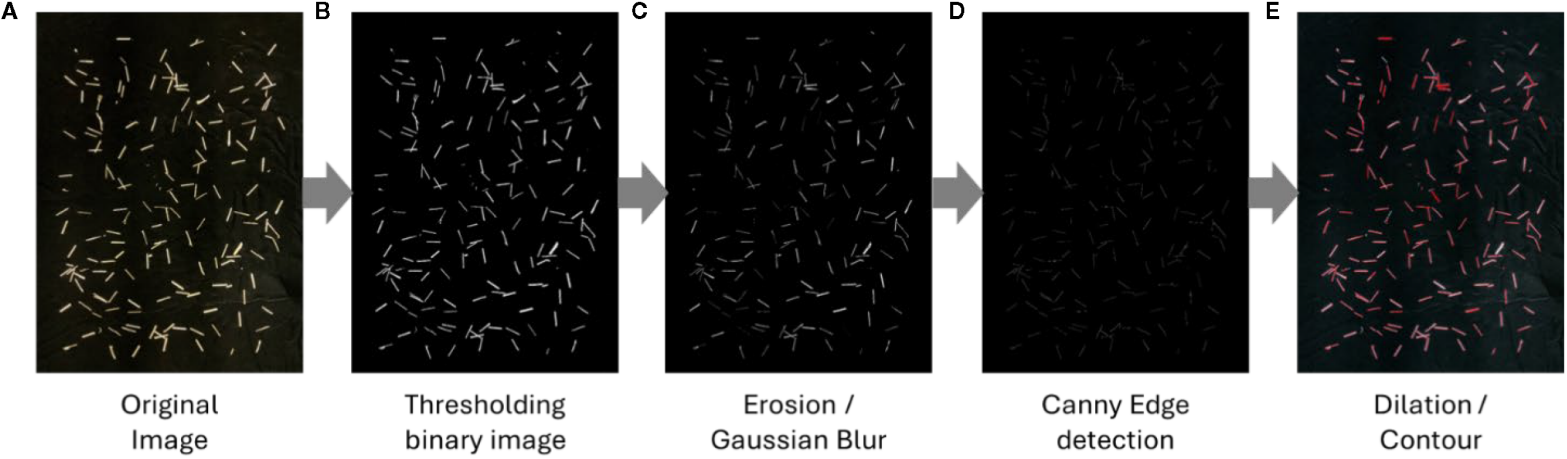
OpenCV transformation workflow for edge-based segmentation. Pipeline panels **(A**–**E)** show: **(A)** original RGB image; **(B)** HSV conversion and hue thresholding to create a binary mask (tillers = white, background = black); **(C)** morphological erosion and Gaussian blur to suppress noise (seeds/dust); **(D)** Canny edge detection to delineate boundaries; **(E)** dilation to strengthen edge continuity before counting. This workflow reduces false positives and prepares masks for baseline estimation.

To establish a classical computer vision baseline for comparison with deep learning models, an edge-based segmentation pipeline was implemented using the OpenCV library (Bradski, 2000). RGB images (**Figure 3A**) were converted to HSV color space and thresholded by hue to generate binary masks in which tillers appeared as white foreground objects against a black background (**Figure 3B**). This transformation enhanced pixel separability and reduced color-related artifacts associated with natural plant variation. Morphological erosion followed by Gaussian blurring was applied to suppress noise arising from small debris and dust particles (**Figure 3C**). Canny edge detection (Canny, 1986) was then used to delineate object boundaries, and dilation was applied to strengthen edge continuity and reduce fragmentation (**Figures 3D, E**). Final tiller counts were derived through connected-component analysis, with each distinct segmented region treated as an individual instance. While computationally efficient and transparent, this pipeline served primarily as a baseline for evaluating improvements provided by convolutional neural network–based detectors under increasing levels of tiller density and occlusion.

### 2.4 Annotation dataset construction and augmentation

A total of 770 images were collected across low, medium, and high tiller-density conditions. To create the annotation dataset, 10% of the full image set (n = 90 images) was randomly selected while preserving proportional representation across density categories to minimize sampling bias. These 90 original images contained 21,603 manually labelled tillers, with bounding boxes generated using LabelMe software. Annotation quality was confirmed by spot-checking randomly selected images for consistency. To increase training diversity, each annotated image underwent horizontal and vertical flipping, 45° rotation, and RGB–HSV color-space conversion. Following augmentation, the number of labeled instances increased from 21,603 to 64,809 annotated tillers. Because each image contained isolated tillers on a uniform black background, the visual complexity of individual scenes was low, and the model learned primarily from the large number of clearly defined labeled objects rather than relying on numerous unique image backgrounds. An additional 6,123 annotations were later incorporated from medium- and high-density images to assess the influence of occlusion-focused labelling. The full annotated dataset was partitioned into 85% training, 7.5% validation, and 7.5% testing subsets. The remaining 90% original of the original 770 images were held out entirely for independent evaluation against manual counts.

### 2.5 Faster R-CNN model training

**Figure 4:**
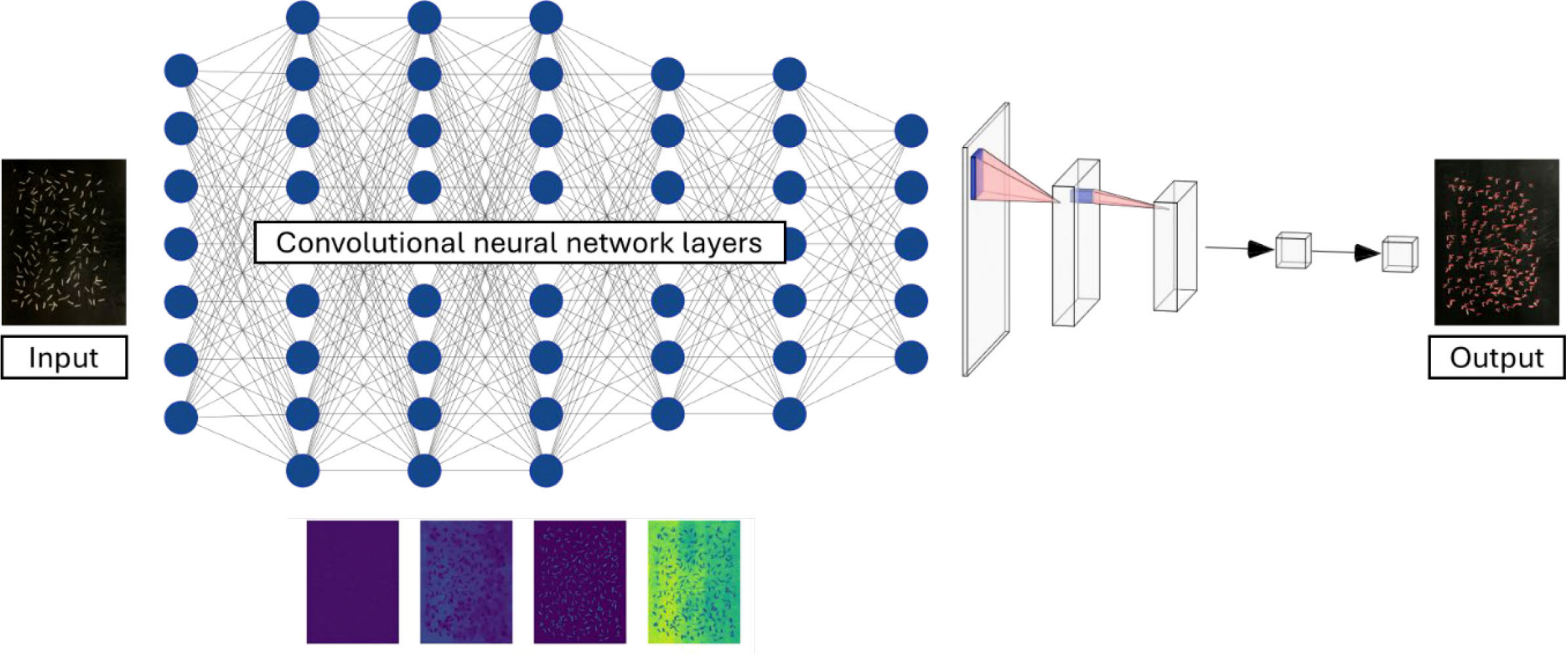
Schematic of CNN feature learning for tiller detection. The input image is processed through successive backbone layers, extracting hierarchical features across channels. Deeper layers encode shape and texture context and support final detection heads that localize and label tillers in crowded scenes.

Both Faster R-CNN (Cai and Vasconcelos, 2018; Ganesh et al., 2019; Lin et al., 2020; Afzaal et al., 2021; Wang et al., 2023) and YOLOv8 (Bochkovskiy et al., 2004; Bai et al., 2023; Wang et al., 2023) models were trained on identical datasets to ensure direct comparability (**Figure 4**). Training was conducted on a high-performance workstation equipped with an Intel^®^ Xeon^®^ Gold CPU, 128 GB RAM, and an NVIDIA RTX A5000 GPU with 24 GB VRAM. Hyperparameters evaluated included backbone architecture (ResNet-50 vs. ResNet-101), input resolution (640 × 640 vs. 1280 × 1280 pixels), and batch size (2 vs. 8). Iteration limits were set to 15,000 for ResNet-50 and 20,000 for ResNet-101 based on stabilization of training and validation loss curves. Fixed random seeds were applied to enhance reproducibility. Detection outputs were converted to final tiller counts using a consistent confidence threshold and non-maximum suppression (NMS) intersection-over-union threshold across all experiments. Model performance was evaluated using precision and mean average precision at 50% intersection-over-union (mAP@50) (Hui, 2018), where a predicted bounding box was considered correct if it overlapped the ground-truth box by at least 50%.

### 2.6 YOLOv8 model training

YOLOv8 models were trained on the same computational system and annotation dataset used for Faster R-CNN to ensure consistency across architectures (**Figure 4**). Two model scales—YOLO-Large and YOLO-Extra-Large—were evaluated to compare trade-offs between inference speed and detection accuracy. Input resolutions and batch sizes were systematically varied, and training epochs were capped at 200 based on stabilization of validation loss curves and convergence trends. The YOLO-Large model provided a balance between computational efficiency and predictive accuracy suitable for high-throughput inference, whereas the YOLO-Extra-Large model prioritized maximal detection accuracy through increased parameter depth. Detection-to-count conversion used identical confidence thresholds and non-maximum suppression (NMS) settings as Faster R-CNN to ensure fair comparison across models. All training runs employed fixed random seeds to minimize stochastic variation and enhance reproducibility.

### 2.7 Increasing annotation density for occlusion evaluation

To evaluate whether additional occlusion-rich annotations could improve detection accuracy, 20 medium- and high-density images were manually labeled, contributing 6,123 new bounding boxes. Both convolutional neural network architectures were retrained using their previously optimized hyperparameters, and performance changes were assessed using both detection metrics and counting accuracy. This experiment was designed to isolate the effect of annotation diversity on instance-level detection under heavy occlusion and to determine whether increased representation of overlapping tillers improved model robustness.

### 2.8 Statistical analysis and model evaluation

Counting accuracy was assessed through linear regression (Montgomery et al., 2021) of predicted versus manual counts, with the coefficient of determination (R²) reported as a measure of goodness-of-fit. In addition to R^2^, regression performance was further evaluated using root mean squared error (RMSE), normalized RMSE (NRMSE), and mean absolute percentage error (MAPE) to quantify overall prediction error magnitude, scale-adjusted error, and relative percentage deviation, respectively. Mean absolute error (MAE) and bias were also calculated to quantify absolute discrepancies and directional tendencies. Detection performance was evaluated using precision and mean average precision at 50% intersection-over-union (mAP@50). For deep learning frameworks implemented in PyTorch, mAP@50 was calculated using torchvision (https://pytorch.org/vision/stable/index.html) or the COCO API [https://github.com/cocodataset/cocoapi (github.com in Bing)] (Lin et al., 2014). For YOLOv8, the ultralytics package [https://github.com/ultralytics/ultralytics/blob/main/docs/en/models/yolov8.md (github.com in Bing)] provided built-in mAP evaluation. To further characterize population-level variation, skewness and kurtosis were calculated to assess distributional symmetry and tail weight, respectively. All statistical analyses were performed in Python using the SciPy statistics module (Virtanen et al., 2020), with moment-based calculations implemented through SciPy’s skew and kurtosis functions (https://docs.scipy.org/doc/scipy-0.14.0/reference/generated/scipy.stats.skew.html; (docs.scipy.org in Bing) https://docs.scipy.org/doc/scipy-1.13.0/reference/generated/scipy.stats.kurtosis.html (docs.scipy.org in Bing)). Regression analyses and precision metrics were computed using scikit-learn (Pedregosa et al., 2011) and PyTorch to ensure reproducibility.

## 3 Results

### 3.1 Edge-based segmentation is accurate at low density bur undercounts under occlusion

**Figure 5:**
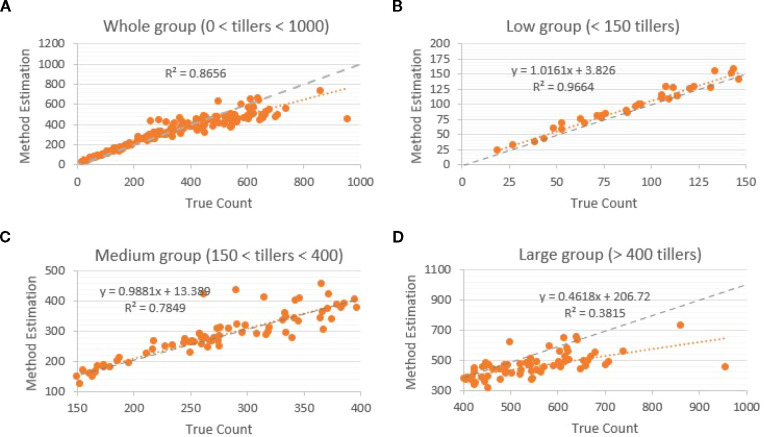
Edge-based segmentation performance by density relative to manual counts. **(A)** Overall regression (R² = 0.86); **(B)** low-density subset (<150 tillers; R² = 0.97); **(C)** medium-density subset (150–400 tillers; R² = 0.78); **(D)** high-density subset (>400 tillers; R² = 0.38). The gray dotted line is the 1:1 identity (y = x). Accuracy declines with occlusion as adjacent tillers merge in binary masks, causing underestimation at higher counts.

We first established a classical computer vision baseline using an edge-based segmentation pipeline and evaluated its performance across the full range of tiller densities present in the dataset. When all samples were pooled, predicted counts showed a strong linear relationship with manual counts(R² = 0.86; **Figure 5A**) indicating the method performed well under uncongested conditions. However, stratification by tiller density revealed substantial variation in accuracy. In low-density images containing fewer than 150 tillers, the method achieved excellent agreement with ground truth (R2 = 0.97; **Figure 5B**), with predicted values tightly following the 1:1 identity line. Performance decreased in the medium-density range of 150–400 tillers, where overlapping structures began to merge in the binary masks; in this subset, the regression fit dropped to R² = 0.78 (**Figure 5C**), reflecting increasing difficulty in separating adjacent tiller bases. The decline was most pronounced in high-density images containing more than 400 tillers, where heavy occlusion consistently caused multiple tillers to be segmented as a single object. In this group, predicted counts showed substantial underestimation relative to manual counts, with R² falling to 0.38 (**Figure 5D**). Across all panels of **Figure 5**, the gray dotted line representing the 1:1 identity (y = x) highlights the density-dependent divergence between predicted and true values, particularly beyond approximately 400 tillers per image.

Visual inspection confirmed that the primary source of error was the merging of highly overlapping tillers into single contours, leading to systematic and increasingly severe undercounting in crowded scenes.

### 3.2 CNN-based detection: architectural trade-offs and performance by density

#### 3.2.1 Faster R-CNN emphasized localization precision but loses count accuracy at high density

**Figure 6:**
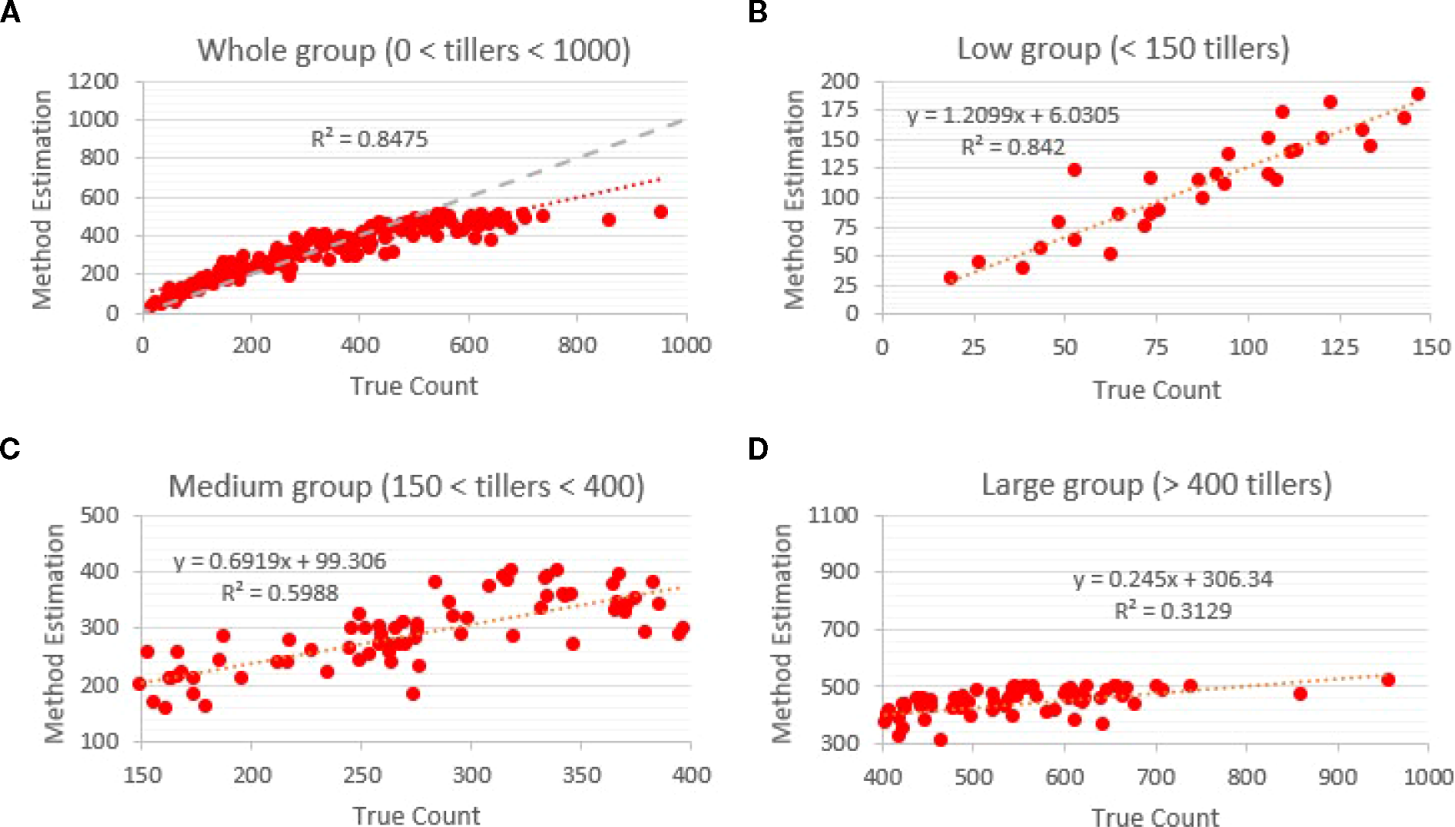
Faster R-CNN count accuracy by density relative to manual counts. **(A)** Overall regression (R² = 0.85); **(B)** low-density (<150 tillers; R² = 0.84); **(C)** medium-density (150–400; R² = 0.60); **(D)** high-density (>400; R² = 0.31). The gray dotted line is the 1:1 identity (y = x). Despite strong mAP@50, predicted totals diverge from the identity line as density increases, reflecting proposal saturation and occlusion limits.

Training configurations and localization performance for Faster R-CNN are summarized in **Table 1**. Two backbone-resolution combinations were evaluated: (i) a ResNet-50 FPN model trained on 640 × 640 px images with a batch size of 8 for 15,000 iterations, and (ii) a ResNet-101 FRN model trained on 1280 × 1280 px images with a batch size of 2 for 20,000 iterations. Both models were pretrained, and Adam was used as the optimizer. Among these configurations, the higher-capacity ResNet-101 FPN at 1280 × 1280 achieved the best localization precision, reaching an mAP 50of 0.85, compared with 0.83 for the ResNet-50 FRN model =. Despite these strong detection metrics, regression of predicted versus manual counts revealed considerable density-dependent variation in total-count accuracy. When evaluated across all images, Faster R-CNN achieved an overall R² of 0.85 (**Figure 6A**). Accuracy remained high in low-density images containing fewer than 150 tillers, where predicted counts closely tracked the 1:1 identity line and the regression fit reached R² = 0.84 (**Figure 6B**). However, performance decreased in medium-density scenes (150–400 tillers), where overlapping tillers reduced separability and the regression fit declined to R² = 0.60 (**Figure 6C**). The reduction in accuracy was most pronounced in high-density images containing more than 400 tillers; in this subset, the model substantially underestimated tiller number, with the regression fit decreasing to R² = 0.31 (**Figure 6D**).

**Table 1:** Faster R-CNN training configurations and localization precision (mAP@50).

Model	Pretrained	Input Image resolution (pixels)	Batch-size	Iteration	Optimizer function	Best mAP@0.5
Faster R-CNN ResNet50-FRN	Yes	640x640	8	15,000	Adam	0.83
Faster R-CNN ResNet101-FRN	Yes	1280x1280	2	20,000	Adam	0.85

Backbones (ResNet-50, ResNet-101), input resolutions (640 × 640; 1280 × 1280 px), batch sizes (2; 8), and iteration counts (15,000; 20,000). Best mAP@50 in bold. The ResNet-101, 1280 × 1280, batch size = 2 configuration achieved mAP@50 = 0.85, outperforming ResNet-50 (mAP@50 = 0.83).

Across all density groups, the gray dotted line representing the identity relationship (y = x) highlights how predicted totals diverged from true counts as tiller density and occlusion increased. Faster R-CNN detections were converted to counts using a 0.5 confidence threshold and a 0.7 IoU threshold for non-maximum suppression, and the full training procedure required approximately 26 hours. These results show that while Faster R-CNN achieved comparatively high localization precision, its proposal-driven architecture struggled to maintain accurate total counts under moderate to severe occlusion.

#### 3.2.2 YOLOv8 prioritizes recall and delivers the highest count accuracy with practical throughput

Training configuration and localization performance for YOLOv8 are summarized in **Table 2**. Two model scales (YOLOv8-L and YOLOv8-X) were evaluated across two input resolutions (640 × 640 px and 1280 × 1280 px), each trained for 200 epochs using the Adam optimizer. The YOLOv8-X model trained on 1280 × 1280 px images with a batch size of 2 achieved the highest localization precision, with mAP@50 = 0.75, whereas the YOLOv8-L configuration trained on 640 × 640 px.

**Table 2:** YOLOv8 training configurations and localization precision (mAP@50).

Model	Pretrained	Input Image resolution (pixels)	Batch-size	Epoch	Optimizer function	Best mAP@0.5
YOLOv8(l)	Yes	640x640	8	200	Adam	0.71
YOLOv8(x)	Yes	640x640	8	200	Adam	0.72
YOLOv8(x)	Yes	1280x1280	2	200	Adam	0.75

**Figure 7:**
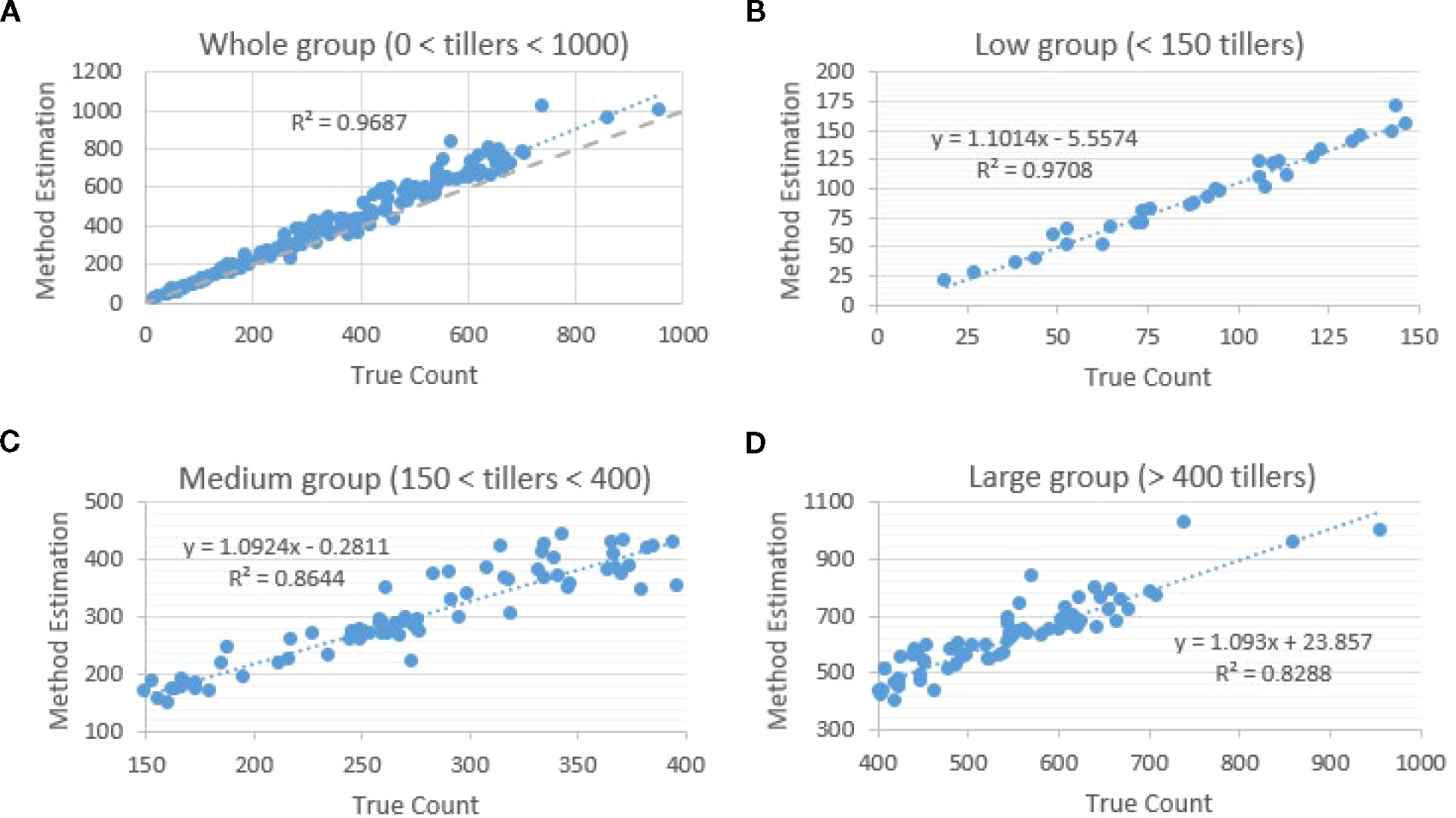
YOLOv8 count accuracy by density relative to manual counts. **(A)** Overall regression (R² = 0.97); **(B)** low-density (<150 tillers; R² = 0.97); **(C)** medium-density (150–400; R² = 0.86); **(D)** high-density (>400; R² = 0.83). The gray dotted line is the 1:1 identity (y = x). YOLOv8 maintains near-identity tracking across densities, reflecting higher recall and superior total estimation in crowded scenes.

Model scales (YOLO-L; YOLO-X), input resolutions (640 × 640; 1280 × 1280 px), batch sizes (2; 8), and epochs (200). The default optimizer was Adam for stable convergence. Best mAP@50 in bold. The YOLO-X, 1280 × 1280, batch size = 2 configuration achieved mAP@50 = 0.75, while YOLO-L, 640 × 640, batch size = 8 produced mAP@50 = 0.71–0.72.

images with batch size 8 produced mAP@50 values ranging from 0.71 to 0.72. Although these localization values were lower than those observed for Faster R-CNN, YOLOv8 achieved markedly superior counting accuracy across all density levels. When evaluated across the full dataset, YOLOv8 predictions showed an overall regression fit of R² = 0.97 relative to manual counts (**Figure 7A**). Accuracy remained similarly strong in low-density images (<150 tillers), where R² reached 0.97 and predictions closely tracked the 1:1 identity line (**Figure 7B**). Performance remained high in medium-density scenes (150–400 tillers), with R² = 0.86 (**Figure 7C**), and declined only modestly in high-density images (>400 tillers), where occlusion is most severe, yielding an R² of 0.83 (**Figure 7D**).

Across all panels, the gray dotted identity line highlights the close agreement between predicted and true counts, illustrating YOLOv8’s ability to maintain high recall and stable estimation even in crowded scenes where overlapping structures commonly reduce separability.

Both YOLOv8 and Faster R-CNN models used identical detection-to-count conversion settings (confidence threshold = 0.5; NMS IoU threshold = 0.7), yet training time differed substantially: YOLOv8 completed training in ~4 hours, compared with ~26 hours for Faster R-CNN. These results demonstrate that YOLOv8’s one-stage, recall-oriented architecture not only provides practical computational efficiency but also delivers the highest overall counting accuracy among all evaluated methods.

### 3.3 Effects of adding occlusion-rich annotations: selective gains and density-dependent trade-offs

To evaluate whether expanding the annotation set could improve detection performance under heavy occlusion, we incorporated 6,123 additional bounding boxes from medium- and high-density images and retrained both YOLOv8 and Faster R-CNN using their previously optimized hyperparameters. The adjusted training configurations and changes in localization precision are summarized in **Table 3**.

**Table 3:** Effect of adding occlusion-rich annotations on training configurations and mAP@50.

Model	Pretrained	Input Image resolution (pixels)	Batch-size	Epoch/ Iteration	Optimizer function	Best mAP@0.5
YOLOv8(x)	Yes	1280x1280	2	200	Adam	0.79
Faster R-CNN ResNet101-FRN	Yes	1280x1280	2	20,000	Adam	0.86

Parameters used to retrain YOLOv8 and Faster R-CNN after integrating 6,123 additional annotations from medium and high-density images; best mAP@50 in bold. mAP@50 improved modestly (YOLOv8: 0.75 → 0.79; Faster R-CNN: 0.85 → 0.86). Density-specific changes in count accuracy are detailed in **Figure 5**.

**Figure 8:**
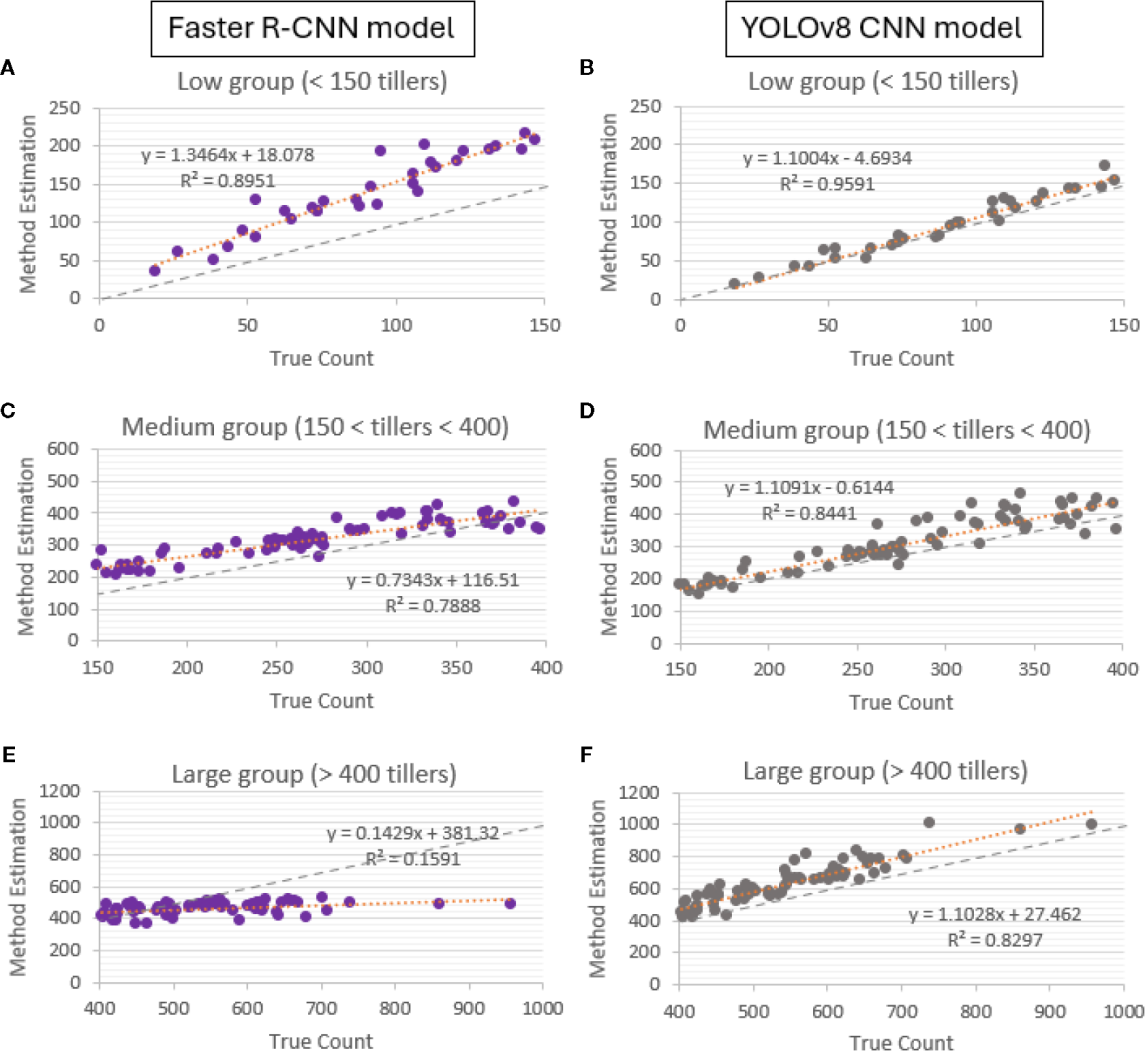
Post-annotation count accuracy by density for Faster R-CNN and YOLOv8. **(A**, **C**, **E)** Faster R-CNN regressions for low (<150), medium (150–400), and high (>400) density groups; **(B**, **D**, **F)** corresponding YOLOv8 regressions. The gray dotted line is the 1:1 identity (y = x). Faster R-CNN improved substantially at medium density (R²: 0.60 → 0.79), declined at high density (R²: 0.31 → 0.16), and slightly overestimated in low density (R²: 0.84 → 0.89). YOLOv8 showed limited changes in R², indicating that added occlusion labels increased mAP@50 without improving totals under heavy occlusion.

Incorporating the additional occlusion-rich labels produced modest gains in mAP@50 for both architectures (YOLOv8: 0.75 → 0.79; Faster R-CNN: 0.85 → 0.86), indicating improved boundary delineation but not necessarily enhanced total-count estimation. Consistent with this, density-specific regression analyses (**Figure 8**) revealed asymmetric effects across architectures. For Faster R-CNN, medium-density performance improved substantially (R² increasing from 0.60 to 0.79), suggesting that additional examples of moderate overlap enhanced proposal distinction in these scenes. However, accuracy declined at high density (R² decreasing from 0.31 to 0.16), and slight overestimation occurred in low-density images (R² increasing from 0.84 to 0.89). In contrast, YOLOv8 exhibited only modest changes in R² across densities (low: 0.97 → 0.96; medium: 0.86 → 0.84; high: 0.83 → 0.83), indicating that while additional occlusion labels increased localization precision, they did not materially affect overall counting fidelity, particularly in the most crowded scenes.

### 3.4 Population-scale distribution and breeding implications

**Figure 9:**
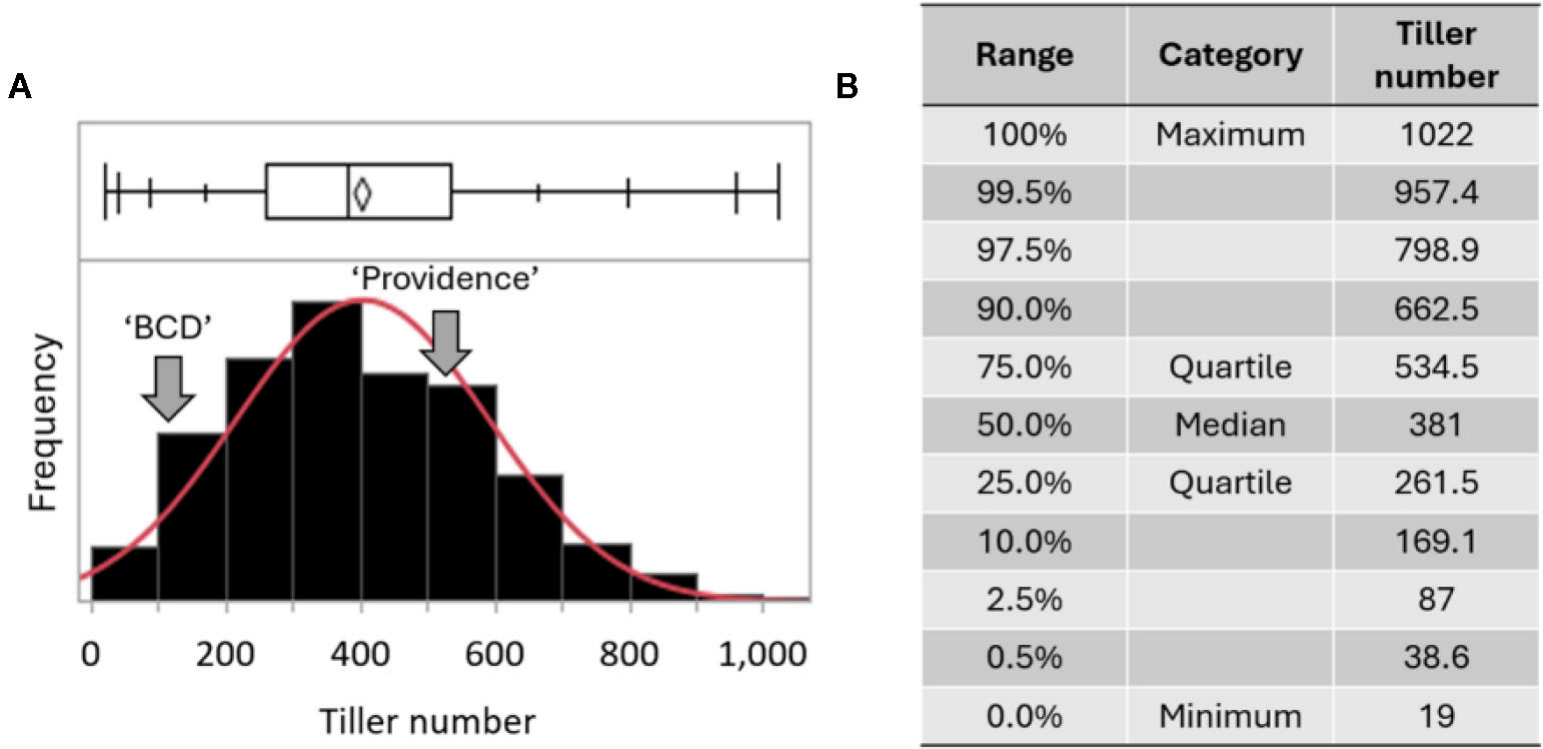
Population-scale distribution of tiller counts and summary statistics. **(A)** Frequency histogram of tiller counts with an overlaid normal density curve (red); **(B)** summary statistics listing minimum (19), maximum (1,022), 25th percentile (261), median (381), and 75th percentile (534). The distribution exhibits near-normal symmetry (skewness = 0.12, kurtosis = 2.64), consistent with quantitative inheritance of tiller number.

Application of the optimized YOLOv8 model across the entire population produced a continuous distribution of predictive tiller counts (Figure). Tiller number per plant ranged from 19 to 1,022, with a median of 381. The 25^th^ and 75^th^ percentiles were 261 and 534, respectively. Distribution metrics indicated slight right skew (skewness = 0.12) and moderate tail weight(kurtosis = 2.64). The overlaid normal density curve aligned closely with empirical frequency histogram, and the corresponding boxplot (**Figure 9A**) and quantifiable table (**Figure 9B**) further summarized population-level variation.

## 4 Discussion

Accurately quantifying tiller number remains a persistent phenotyping challenge in grass breeding because the trait is both biologically important and structurally difficult to measure at scale. In this study, we demonstrate that instance-level tiller counting in bentgrass—defined by thin, filamentous morphology and substantial crown-level occlusion—can be achieved with high accuracy and practical throughput when controlled imaging is paired with a recall-oriented deep learning detector. By systemically comparing a classical edge-based pipeline with two widely used object detection architectures, we clarify the methodological trade-offs among counting fidelity, localization precision, and computational efficiency—trade-offs often obscured when evaluations rely solely on detection metrics. Our aim is not to replace aerial phenotyping, but to establish a precision-oriented measurement layer capable of delivering the instance-level accuracy required for quantitative genetic analysis.

The edge-based segmentation baseline demonstrated that classical methods remain computationally efficient and effective under minimal occlusion, but they fail in dense, small-object environments typical of bentgrass clippings. To understand how modern architectures address these challenges, we next compared Faster R-CNN—a two-stage, proposal-driven detector—and YOLOv8—a one-stage detector optimized for throughput and recall. To contextualize these findings, we discuss how the observed architectural behaviors relate to prior phenotyping work, their implications for throughput and counting accuracy, and the biological and practical consequences for bentgrass breeding.

### 4.1 Relationship to prior image-based phenotyping and the precision-throughput trade-off

Recent advances in computer vision have enabled reliable counting of large and visually distinct plant organs, including as maize tassels, wheat spikes, and field-scale plant stands (Lu et al., 2015; Sadeghi-Tehran et al., 2019; Kumar et al., 2020; Liu et al., 2020; Lu and Cao, 2020; Khaki et al., 2022). These studies highlight the value of deep convolutional neural networks for high-throughput trait extraction. However, most prior applications focus on organs with clear boundaries and strong contrast—conditions that differ substantially from the fine-scale, densely overlapping morphology of bentgrass tillers.

In cereal crops, side-view imaging, LiDAR, and density-map regression approaches have provided useful estimates of tiller number without explicit instance delineation (Singh et al., 2022; Kinose et al., 2022). Yet these strategies do not address the extreme occlusion and instance density encountered at the bentgrass crown. This distinction motivated our focus on the broader precision–throughput spectrum that underpins phenotyping strategies. UAV and canopy-level imaging platforms excel at rapid surveillance, stress detection, and relative genotype ranking across large populations, but lack the spatial resolution needed for organ-level accuracy. In contrast, our controlled instance-level imaging approach prioritizes measurement precision over field-wide coverage—a trade-off that becomes essential when the trait of interest feeds directly into quantitative genetic analysis. For tiller number, measurement error reduces statistical power for quantitative trait locus (QTL) detection and heritability estimation; thus, precision is not merely advantageous but genetically consequential (Jiang and Li, 2020; Das et al., 2019).

Image-based tiller quantification has followed three main paths: classical image processing, 3D sensing, and deep learning detection. Earlier regression-based approaches improved over heuristic pipelines but required controlled backgrounds and did not perform explicit instance separation (Kinose et al., 2023). Field-scale 3D methods, including LiDAR and micro-CT/RGB systems, reconstructed canopy structure but suffered from cost, operational constraints, or modest accuracy under dense occlusion (Fang et al., 2020). More recent work shows YOLO-based detectors outperforming earlier models for small plant structures when attention modules or modified loss functions are added (Liang et al., 2025), and proximal non-DL pipelines have achieved moderate correlations in rice but still face occlusion-driven errors (Yamagishi et al., 2022).

Within this context, our bentgrass workflow delivers explicit base-level detection using YOLOv8 trained on manually annotated images and evaluated across realistic population-level variability. YOLOv8 outperformed both the classical segmentation approach and Faster R-CNN across all density groups, particularly under high occlusion. Extending detection to the entire population revealed a near-normal distribution of tiller counts—information rarely provided in prior tiller-counting studies limited to small datasets or controlled scenes.

### 4.2 Efficiency of controlled imaging and practical throughput

A frequent concern with controlled or semi-destructive imaging is operational efficiency. In our workflow, clipping and staging required roughly one minute per plant, representing a substantial reduction in labor compared with manual separation and counting, which can exceed 10–20 minutes per sample even under ideal conditions. We view this time investment as modest relative to the gains in accuracy and reproducibility. Our approach therefore occupies a pragmatic middle ground: far faster than manual counting yet markedly more precise than canopy-level estimation for fine, filamentous structures.

We also emphasize that the controlled isolation step is not a limitation of the method but a deliberate strategy to reveal true instances in a morphology where UAV viewing angles, shadowing, and pixel resolution compress vertical structure and merge silhouettes. Similar separability challenges have been documented in small-object detection benchmarks in agriculture, where instance isolation or structural priors often determine success more than model depth alone (Allmendinger et al., 2025; Sharma et al., 2024).

### 4.3 Architecture trade-offs: one-stage vs. two-stage detection for counting under occlusion

Our comparative results highlight a central insight: localization precision (mAP) and counting accuracy are not interchangeable metrics in crowded scenes, and architectural design strongly influences this relationship. Faster R-CNN, a two-stage detector, achieved higher mAP@50 but produced less reliable counts at medium and high densities. YOLOv8, a one-stage detector, delivered substantially higher counting accuracy despite slightly lower mAP. This pattern reflects a fundamental difference between the two paradigms: Two-stage detectors prioritize bounding-box precision through region proposals but suffer proposal saturation and missed detections when many overlapping small objects compete for limited proposals, whereas one-stage detectors prioritize recall by directly predicting dense grids of candidate boxes, enabling them to capture more true instances even when boundaries are less precise.

For counting tasks involving small, overlapping objects, minimizing false negatives is more consequential than achieving perfectly tight bounding boxes. In heavily occluded settings, slightly noisier boxes that capture more true tillers yield more accurate totals than precise boxes that miss them. Our findings align with prior observations that single-stage detectors often outperform two-stage models in real-time or small-object agricultural applications (Allmendinger et al., 2025; Sharma et al., 2024), and extend this logic to the extreme occlusion characteristic of bentgrass tillers.

Our classical edge-based segmentation (thresholding + contours) provided a transparent and computationally efficient baseline, but it systematically undercounted as canopy density increased—in our dataset, RMSE rose from 8.45 in small groups to 137.79 in large groups, and R² declined from 0.97 to 0.38 (**Table 4**). This density-related degradation is consistent with agricultural reports showing that classical image-processing methods break down under occlusion and overlap in dense cereal canopies, where adjacent organs merge into single contours and precision and recall deteriorate (Wang et al., 2021). Recent journal studies therefore emphasize recall-oriented deep detectors designed for small, dense, and overlapping targets, demonstrating improved detection and counting accuracy across planting densities, growth stages, and UAV imaging scenarios (Yao et al., 2022; Yang et al., 2021). In line with these findings, our YOLOv8 model maintained higher accuracy and stronger model fit than the classical baseline across population-level variability, supporting its suitability for instance-level tiller counting in crowded bentgrass canopies.

**Table 4:** Comparative prediction performance of OpenCV, YOLOv8, and Faster R-CNN for tiller detection across small, medium, and large group sizes.

Group Size	Count range	OpenCV				YOLOv8				Faster R-CNN			
		R^2^	RMSE	NMRSE	MAPE	R^2^	RMSE	NMRSE	MAPE	R^2^	RMSE	NMRSE	MAPE
Small	128	0.97	8.45	6.60%	8.46%	0.97	6.65	5.19%	6.08%	0.84	52.92	41.35%	59.40%
Medium	247	0.78	32.38	13.11%	7.96%	0.86	35.59	14.41%	10.25%	0.59	53.04	21.48%	19.83%
Large	553	0.38	137.79	24.92%	17.91%	0.83	83.33	15.07%	13.16%	0.31	148.13	26.79%	16.51%

Metrics include coefficient of determination (R²), root mean square error (RMSE), normalized RMSE (NRMSE), and mean absolute percentage error (MAPE), calculated relative to manually annotated ground-truth tiller counts.

### 4.4 Biological relevance and breeding implications

Automated generation of accurate, population-scale tiller counts provides access to quantitative trait analyses that were previously constrained by the time and labor required for manual measurements. When applied across the entire interspecific hybrid population, the optimized YOLOv8 model produced a continuous and near-normal distribution of tiller numbers, ranging from 19 to 1,022 per plant. The slight right skew (0.12) and moderate kurtosis (2.64) reflect the presence of high-tillering individuals and are consistent with quantitative inheritance patterns characteristic of complex agronomic traits. This distribution offers a robust phenotypic foundation for downstream analyses, including QTL mapping and marker-assisted selection.

The model-derived trait values aligned with field observations, where plants with higher predicted tiller counts exhibited visually denser canopy structure. Because tillering capacity influences canopy density, biomass accumulation, and seed yield potential, the ability to quantify this trait at high resolution and scale enables more informed selection decisions. Integrating these phenotypes with additional agronomic traits will help identify alleles associated productivity, resilience, and adaptation in bentgrass breeding programs.

The computational efficiency achieved with YOLOv8 also allows this phenotyping layer to be incorporated into routine seasonal evaluation cycles without causing delays. By narrowing the gap between genotyping throughput and phenotyping capacity, this workflow supports the integration of precise, high-volume trait measurements into modern breeding pipelines (Jiang and Li, 2020; Das et al., 2019). In this context, accurate instance-level tiller counts represent not only an improvement in measurement precision but also a practical enabler of accelerated genetic gain.

### 4.5 Limitations and future directions

Several limitations define the operational scope of our workflow. First, performance is optimized for controlled imaging following clipping preparation; separability is expected to decrease in unconstrained field scenes where illumination, soil background, and canopy occlusion vary substantially. Second, instance-level detectors still struggle at the highest densities, where overlapping structures exceed separability limits and post-processing steps such as non-maximum suppression may remove valid detections. Third, annotation remains a meaningful investment for small, crowded objects, and simply increasing the number of labels does not necessarily translate into proportional gains in counting accuracy.

Looking ahead, several opportunities exist to address these constraints. Instance segmentation approaches (mask-level outputs) may improve separation of overlapping tillers (He et al., 2017; Ultralytics, 2023), and structure-aware supervision—such as skeleton or centerline priors—could help reduce false negatives in filamentous objects (Xie et al., 2021). Hybrid detection–density objectives may also stabilize totals in the highest-density regime, where aggregate signals exceed instance-level resolvability (Li et al., 2018). In addition, multi-view imaging and weakly supervised annotation strategies could reduce labeling burden while maintaining count fidelity.

Conceptually, we view a two-tier pipeline—UAV-based screening followed by instance-level precision quantification—as a practical way to balance speed and accuracy without expecting a single modality to satisfy both needs. This layered approach aligns with the broader precision–throughput framework that increasingly guides phenotyping system design.

## 5 Conclusion

In summary, our study demonstrates that accurate and high-throughput tiller quantification in bentgrass is achievable when controlled instance-level imaging is paired with a recall-oriented deep learning detector. Through systemic comparisons of classical segmentation, a two-stage detector, and a one-stage detector, we should that architectural design strongly influence counting accuracy under severe occlusion. In particular, YOLOv8 consistently delivered the most reliable total count across density regimes, even though it exhibited lower localization precision than Faster R-CNN, highlighting that recall and robustness to overlap are more critical than bounding-box tightness when quantifying densely packed, filamentous structures. model for counting under severe occlusion, even when localization precision is lower. By positioning this workflow as a precision complement to aerial surveillance rather than a replacement, we address a practical phenotyping bottleneck and provide an approach that can be adapted to other grass species where tillering is agronomically relevant. Although our focus is intentionally narrow—emphasizing reproducibility and practical throughput over broad field generalization—the ability to integrate these counts into quantitative genetics and selection pipelines makes the workflow a valuable component of modern breeding programs.

## Data Availability

The original contributions presented in the study are included in the article/supplementary material. Further inquiries can be directed to the corresponding author.
